# La pancréatite aiguë: un mode de révélation rare du myélome multiple

**DOI:** 10.11604/pamj.2014.17.313.4340

**Published:** 2014-04-25

**Authors:** Tarik Souiki, Illiassou Soumeila

**Affiliations:** 1 Service de Chirurgie Viscérale, CHU Hassan II, Fès, Maroc; 2 Service de Néphrologie, CHU Hassan II, Fès, Maroc

**Keywords:** Pancréatite aiguë, myélome multiple, protéinurie de Bences jones, acute pancreatitis, multiple myeloma, Bences jones proteinuria

## Image en medicine

Les pancréatites aigues restent majoritairement d'origine biliaire dans notre contexte. Les autres étiologies sont rarement identifiées et la pancréatite est le plus souvent classée « idiopathique ». Nous rapportons un cas de pancréatite aigue révélant un myélome multiple, en insistant sur l'intérêt des paramètres biologiques et de l'imagerie dans l'orientation diagnostique. Notre patient est âgé de 45 ans, ayant des antécédents de coliques néphrétiques épisodiques non documentées, a été admis pour des épigastralgies transfixantes évoluant depuis 3 jours dans un contexte d'apyrexie. A l'examen clinique, le patient présentait une sensibilité épigastrique isolée. Le bilan biologique objectivait une hyperlipasémie à 17 fois la normale. Le scanner abdominal objectivait une pancréatite stade C: pancréas augmenté de taille avec infiltration de la graisse péri-pancréatique. Par ailleurs, on note de multiples lésions ostéolytiques vertébrales et une lithiase calicielle droite (A). Les explorations biologiques montrent une hypercalcémie à 123 mg/l, une insuffisance rénale à 22 ml/min de clearance de créatinine. Le dosage de PTH était par ailleurs normal. Un traitement d'urgence de cette hypercalcémie majeure a été instauré à base d'une hyper-hydratation intraveineuse associée à la corticothérapie. Une radio du crane de face avait mis en évidence de multiples lésions ostéolytiques à « l'emporte pièces ». L'association de telles lésions ostéolytiques oriente l'enquête étiologique de l'hypercalcémie vers un mécanisme de déminéralisation osseuse. L’électrophorèse des protéines urinaires avait identifié une protéinurie de Bences jones. L'immunofixation des protéines plasmatiques et urinaires avait mis en évidence la présence d'une gammapathie monoclonale à chaine légère type kappa. Le myélogramme a révélé une infiltration plasmocytaire à 35% confirmant le diagnostic de myélome multiple. Une chimiothérapie a été alors démarrée.

**Figure 1:**
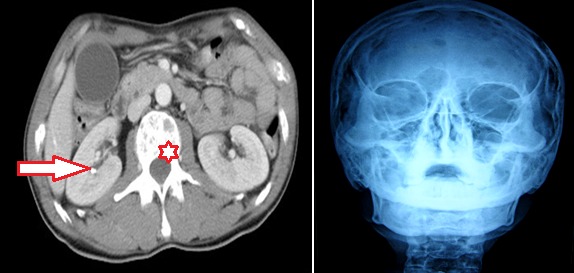
A) Coupe scanographique abdominale (L2): montrant une pancréatite stade C, des lésions ostéolytiques du corps vertébral (astérisque) et une lithiase calicielle (flèche); B) radiographie de crane de face montrant des lésions ostéolytiques à « l'emporte pièce »

